# PolyJet 3D-printed microfluidic device that integrates hydrodynamic focusing of red blood cells and detection of ATP release

**DOI:** 10.1039/d6ay01598k

**Published:** 2026-09-18

**Authors:** Mariama Singhateh, Samuel Azibere, Randy S. Sprague, Ajith Karunarathne, Sashima Liyanage, R. Scott Martin

**Affiliations:** a Department of Chemistry, Saint Louis University 3501 Laclede Ave St. Louis MO 63103 USA scott.martin@slu.edu +1 314-977-2836; b Department of Pharmacological and Physiological Science, Saint Louis University 1402 S. Grand Boulevard Saint Louis MO 63103 USA; c Center for Additive Manufacturing, Saint Louis University USA

## Abstract

In this paper, a PolyJet 3D printed microfluidic device was designed and fabricated (using a print-pause-print technique with liquid support) to hydrodynamically focus and deform red blood cells (RBCs) and also quantitate the release of adenosine triphosphate (ATP) as a function of the deformation forces. Average focusing widths of 70 µm and 28 µm were achieved by constricting the RBCs into well-defined narrow streams at flow rates of 10 µL min^−1^ and 30 µL min^−1^, respectively. Near real-time measurements of ATP release after focusing were carried out *via* a chemiluminescence reaction by introducing luciferin/luciferase mixture into the sheath flow side channels, mixing with any released ATP, and the resulting emitted light was captured with a PMT under the device. The dimensions of the 3D printed focusing/mixing channel were optimized downstream to increase the residence time of the RBCs over the PMT, leading to improvements in calibration sensitivity and the limit of detection for both flow rates. The optimized device was used to detect the ATP release from RBCs as a function of differing focusing widths. Finally, the effect of inhibiting ATP release from RBCs was also determined using diamide and glibenclamide. This PolyJet 3D printing approach clearly provides a method to modulate deformation forces on RBCs and measure the subsequent ATP release in near real-time.

## Abstract 2

3D printed microfluidic device was designed and fabricated to hydrodynamically focus red blood cells and quantitate the release of ATP.

## Graphical abstract

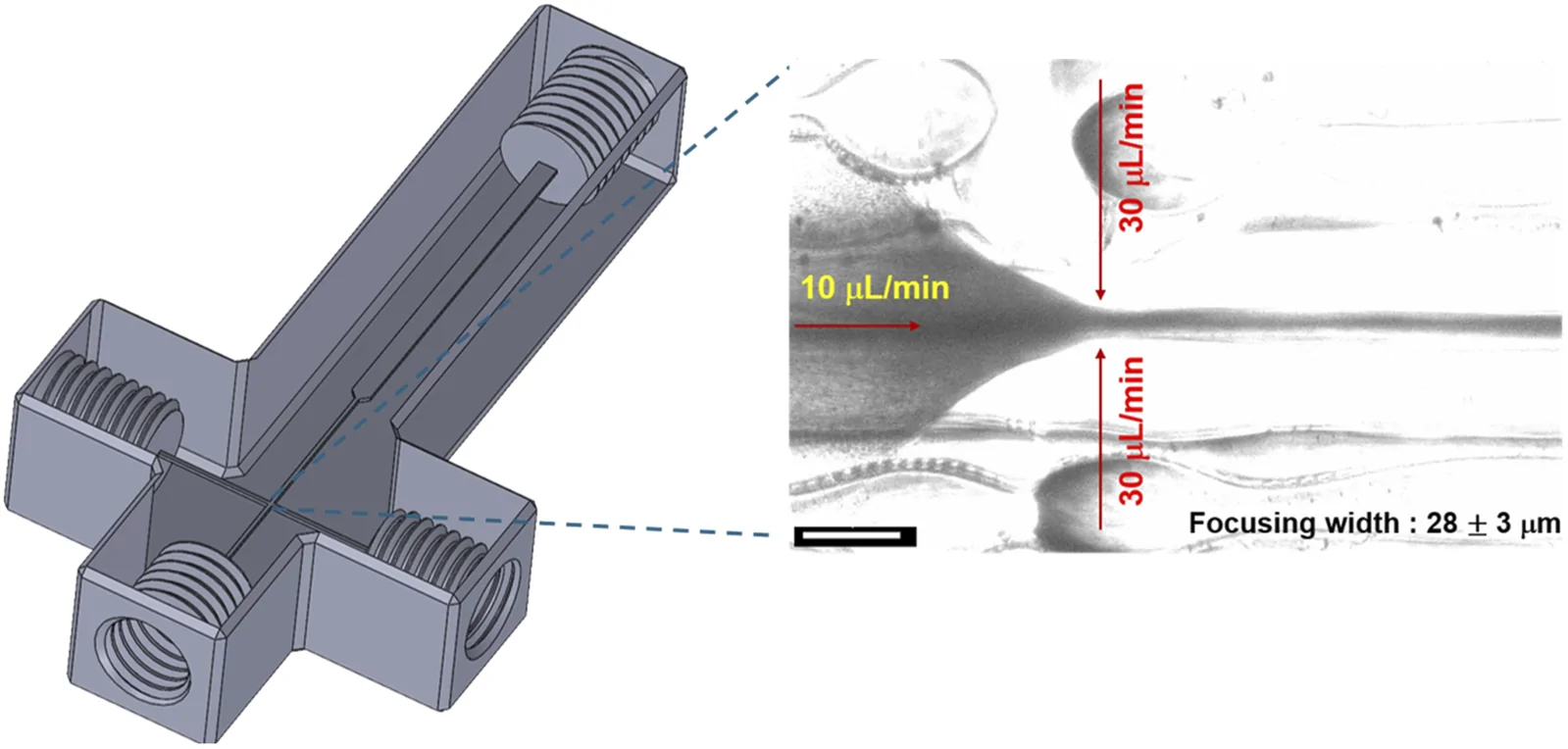

## Introduction

Red blood cells (RBCs) are the most abundant cell type *in vivo* and play a critical role in oxygen and carbon dioxide transport as well as in regulating vascular tone through the release of adenosine triphosphate (ATP).^1^ RBCs are naturally flexible and as they transverse through the circulatory system can deform from their natural biconcave disk shape to pass through capillaries as small as 2–3 microns in diameter.^2^ Sprague *et al.* showed that when RBCs are exposed to reduced oxygen tension or are subjected to mechanical deformation as occurs *in vivo*, a signal transduction pathway is activated that results in the release of the vasodilator, ATP. This pathway involves activation of the heteromeric Gα_i_, which subsequently activates adenyl cyclase, leading to an increase in intracellular cyclic adenosine monophosphate (cAMP).^3^ This is a critical step in the signaling cascade, which culminates in the activation of protein kinase A (PKA) and then phosphorylation of the cystic fibrosis transmembrane conductance regulator (CFTR) protein. Activation of this pathway results in the opening of the pannexin-1 channel, the actual conduit for ATP to exit the cell. Extracellular ATP can then act on endothelial cell surface purinergic receptors, stimulating nitric oxide (NO) production. This NO can then diffuse into the vascular smooth muscle, resulting in cyclic guanosine monophosphate (cGMP) production that promotes vasodilation to match tissue oxygen demand.^4^

Alterations in RBC deformability and biochemical signaling are implicated in various pathological states in humans, including sickle cell disease, diabetes mellitus, malaria, and pulmonary hypertension.^4,5^ In disease states such as diabetes mellitus, RBCs lose flexibility, exhibit increased aggregation, and trigger a pro-inflammatory signaling cascade leading to impaired oxygen delivery and vascular complications.^4^ Moreover, altered RBCs may also play a role in multiple sclerosis (MS), through impaired antioxidant capacity and altered haemorheological properties.^6,7^ In addition, a study by the Spence group showed that MS RBCs release significantly more ATP than normal RBCs under shear stress.^8^ Using a microfluidic model, they demonstrated that steroid-inhibition of RBC-derived ATP could mediate vascular function, highlighting a potential mechanism linking MS treatment to altered blood flow regulation.^8^

Because ATP release is closely linked to RBC deformability and microvascular flow regulation, quantitative measurement of this response has become increasingly important in understanding both normal physiology and associated RBC dysfunction in diseases such as MS and diabetes.^4,5^ Microfluidic technologies provide a powerful platform for investigating RBC deformation^9^ and ATP release under physiologically relevant flow conditions. Unlike conventional bulk assays, microfluidic systems allow precise control of channel geometry, shear stress, and flow dynamics while enabling analysis in close to real time. Initial work in this area involved the use of PDMS-based devices, where RBCs were infused through channels of decreasing size and the released ATP was measured *via* chemiluminescence after on-chip mixing of the flowing RBCs with luciferin/luciferase.^10,11^ It was later shown that hydrodynamic focusing of cells could be used to deform RBCs and integrate chemiluminescence detection of ATP.^12^ Subsequent work from Stone's group showed that ATP release from RBCs occurs rapidly following deformation, with millisecond-scale activation kinetics that directly correlate with shear exposure.^13^

Initially, these and other studies using microfluidic devices to deform RBCs^14,15^ used PDMS-based approaches for the chip fabrication. More recently, 3D printing has become a popular alternative to creating microfluidic devices. This is due to the advantages that 3D printing has over PDMS approaches including reduced fabrication time, the ability to share CAD designs with other researchers,^16^ the ability to produce 3D architectures in devices that are ready to use after printing (eliminating bonding steps),^17^ and the robust fluidic connections that are possible. However, 3D printing with approaches that require solid support layers for channel formation often suffer from poorer resolution with channel sizes that can be 500 µm or larger.^16^ Here, we utilize a print-pause-print technique with PolyJet 3D printing and liquid support^18^ to produce a hydrodynamic focusing device with channels that are 240 µm (average width) by 170 µm (average depth). By varying the flow rate of the side channel focusing streams, we can focus and differentially deform the incoming RBC stream from 70 µm to 28 µm by simply changing the flow rate. The focusing stream contains a luciferin/luciferase solution that mixes with released ATP, producing a chemiluminescent signal that is measured by a PMT placed under the device. The device design was optimized to ensure proper mixing and reaction time. Characterization included evaluating the sensitivity and limit of detection for ATP quantitation as well as monitoring the flow profile with confocal microscopy. Finally, the device was utilized to analyze the ATP release from healthy RBCs using the method of standard additions, with a significant difference in the measured ATP concentrations between the two different focusing widths. This 3D printing leads to a robust device that can be used to quantitatively measure ATP release under different focusing conditions.

## Experimental

### Materials

VeroClear resin and solid support material (SUP706) for the Stratasys J735 printer was obtained directly from Stratasys (Eden Prairie, MN). Fluorescein, ATP, crude firefly lantern, tris(hydroxymethyl)-aminomethane, diamide, glybenclamide, glycerol, and phosphate-buffered saline (10×) were obtained from Sigma-Aldrich (St. Louis, MO). CaCl_2_, NaCl, MgSO_4_, KCl, isopropanol, were obtained from Oakwood Chemicals (USA). Luciferin was obtained from Gold Biotechnology (St. Louis, MO). Glucose was obtained from Fisher Scientific (Fair Lawn, NJ). Bovine serum albumin was obtained from MP Biomedical (Irvine, CA).

### 3D printed microfluidic device fabrication and experimental setup

**Fig. 1:**
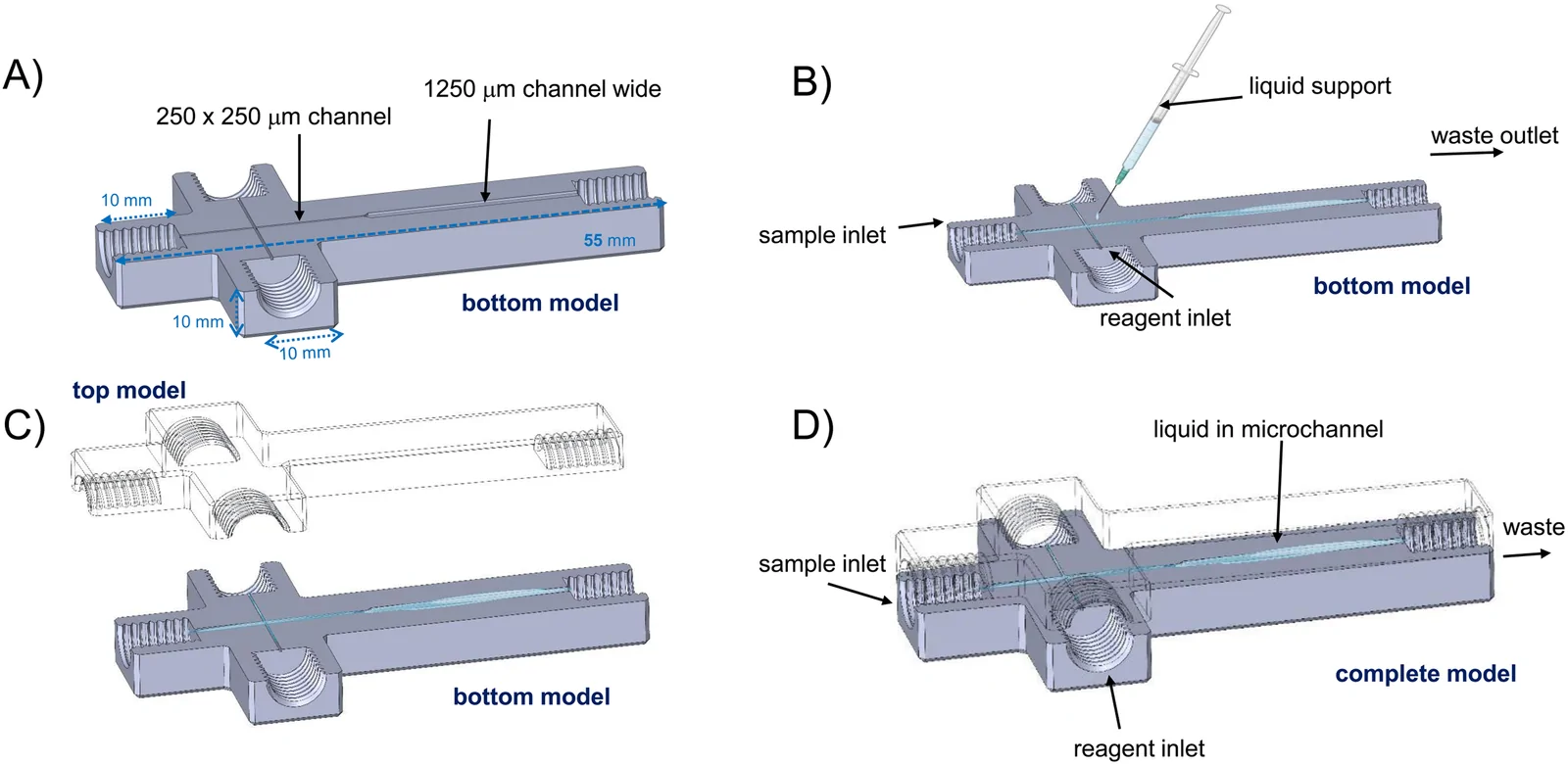
Schematic of print-pause-print PolyJet printing devices with liquid support (a mixture of 65 : 35 v/v glycerol and isopropanol). (A) The bottom model is printed with the channel features open (no solid support). (B) The printer is paused for the liquid support to be added to the channels. (C) The printer is resumed for top model to be printed on top of the bottom model with the channels filled with liquid support. (D) Complete model, with liquid support being removed by pressurizing the device post printing.

A T design was adopted to both focus the RBCs and mix with the luciferin/luciferase reagent. A two-part T-shaped microfluidic device was designed in SolidWorks, with the CAD drawing having a designed 250 µm (depth) × 250 µm (width) channel cross-section around the T-junction and the downstream expansion/mixing channel (which starts 10 mm downstream from the T-junction) being designed with a cross-section of 250 µm (depth) × 1250 µm (width) (Fig. 1). Fluidic connections were made with printed threads designed to accommodate commercial fittings (Idex P-202, IDEX Health & Science, Oak Harbor, WA) for adapters either to tubing for reagents (508 µm i.d. Tygon tubing, 06419-01, Cole-Parmer, Vernon Hills, IL) or capillary for the sample inlet (150 µm i.d. and 360 µm o.d., Polymicro, Phoenix, AZ). The .SLDPRT file was sent to the Stratasys J375 printer (Eden Prairie, MN) and printed with VeroClear material.^19^ A 3D-rendered graphical illustration of the key dimensions of the device is provided in Fig. S1.

The bottom model of the device (Fig. 1A) consists of the main and side channels, with half of the open-threaded design acting as a stop for the print-pause-print methodology. In contrast, the top model features the other half of the threaded designs to complete the T device and the cover. The final design that results is a T device that includes three inlets to introduce a sample of analyte through the main channel and physiological salt solution (PSS) buffer or luciferin/luciferase reagent through the side channels (for both focusing on the T-junction and adding reagents for the chemiluminescence reaction). Finally, there is an outlet stream that goes to waste (Fig. 1). To ensure that the device is printed as a two-part model, changes were made in SolidWorks to isolate the channel from the whole model, and the smart insert feature on the PolyJet J735 printer software was utilized. PolyJet printing channels below 400 µm without photocurable support requires changing the printing parameters such that the 3D model is split into two independent bodies and the application of the print-pause-print technique.^18^ This is achieved by determining, calculating, and changing printer parameters such as the z-height equivalence of the T device and assigning air void to the channel to prevent the printer from depositing solid support material (SUP706) in the channel. Channels were separated from threads in SolidWorks because it serves as a cavity for the Smart insert feature on GrabCAD Print Pro Software (Stratasys J735), which allows designating the channel as air void on model material tray settings and the whole body as VeroClear (so that the channel is left empty), allowing the application of liquid support.

When printing the bottom part of the device (Fig. 1A), the printer stops to allow the addition of a mixture of 65 : 35 v/v glycerol and isopropanol in the channel with a syringe (Fig. 1B).^18^ Excess liquid support was removed with a squeegee, and a microfiber towel was used to wipe excess liquid on the surface device (to ensure proper adhesion once printing is re-started). The printer is then resumed to complete printing the top half of the model (Fig. 1C), after which the complete model results (Fig. 1D). The print time is under an hour, and post-printing processing involved washing the device in a Super DT3 CleanStation (PM Technologies, Inc., Osseo, MN) that contains a sodium hydroxide/sodium metasilicate solution (pH 9) for 30 minutes to help loosen the SUP706 support material deposited in the threads.^20^ An appropriately size tap was used to chase the internal threads and remove any remaining solid support material. Compressed air is used to clear the channel of liquid support, followed by rinsing with water. To minimize surface roughness and maximize optical transparency of the PolyJet-printed parts, small ridges formed at the base of the photopolymerized VeroClear printed part were systematically smoothed using a sequence of progressively finer silicon carbide (SiC) abrasive paper (Buehler, Lake Bluff, IL) in the presence of deionized water. Initially, P800 grit paper was employed, followed by P1200 grit and, finally, ultra-fine P4000 SiC paper.

### UV-Vis spectroscopy

To determine the transparency of the device, a straight channel was printed in the same manner as the T design. Optical transmittance of the 3D printed straight channel device was measured using an Agilent Cary 60 UV-Vis spectrophotometer (Agilent Technologies, Santa Clara, CA, USA) operated in transmittance mode. Transmission spectra were collected over the wavelength range of 200 nm – 800 nm using a spectral bandwidth of 2.0 nm and a scan speed of 600 nm min^−1^. A 10 mm blank UV quartz cuvette (Fisher Scientific) was used as a reference before each measurement. All measurements were performed at room temperature; the percent transmittance was recorded using Cary WinUV software.

### Imaging of devices

An Olympus inverted fluorescence microscope (1X71) equipped with a 100 W Hg Arc lamp and a Qicam fast digital software (NorPix, Montreal Canada) was used to visualize the continuous flow of RBCs in the channel by increasing the sheathing flow rates.^12^ A 2.5 mL SGE (Sigma Aldrich, St Louis, MO) syringe placed on a single pump (Harvard 11 Plus, Havard Apparatus, Holliston MA) was used to introduce RBCs in the device inlet. Flow for the two-side channel inlets was facilitated *via* a 1 mL SGE syringe (Sigma Aldrich, St Louis, MO) for each that was placed on a dual-syringe pump (Harvard PHD 2000, Harvard Apparatus, Holliston MA). Physiological salt solution (PSS) at pH 7.4 was used as the carrier buffer for continuously focusing the RBCs throughout the narrow channel. The channel width and depth are reported as averages and standard deviation of 16 independently printed devices focusing with either 10 µL min^−1^ or 30 µL min^−1^, respectively. Bright field micrographs of the dissected T devices were taken with a Keyence VHX-500K digital microscope (Japan), and several measurements of the channels depth and width were done on the VHX Measurement software. In addition, for imaging of the channel cross section, an FEI Inspect F50 (Lausanne, Switzerland) was used. Dissected parts were sputter-coated with gold for 30 s (Desk V Standard Sputter Coater, Denton Vacuum LLC, Moorestown, NJ) and then imaged by scanning electron microscopy (SEM). Measurements were determined using ImageJ software.

### 3D fluorescence confocal imaging and analysis

Confocal microscopy of a focused fluorescein solution was also used to characterize the device. The methods, protocols, and imaging parameters were adapted from previously published work.^21–23^ Briefly, experiments were performed using a spinning-disk confocal imaging system (Andor Technology) equipped with a 10×, 0.30 NA oil-immersion objective and an iXon ULTRA 897BV back-illuminated, deep-cooled EMCCD camera. The dynamic focusing of a 10 µM fluorescein solution pumped through the microfluidic channel at 10 µL min^−1^ (central stream) and 30 µL min^−1^ phosphate buffered saline (PBS) solution from the side channels was imaged using 488 nm excitation and 515 nm emission. Z-stack images were collected using an ASI-XYZ Automated Stage with a Piezo *Z*-Axis Top Plate, which moved the device 0.75 µm after each image capture, resulting in 300 images along the *Z* axis. The 3D multi-TIF image stack was analyzed using Andor iQ 3.2 software and Imaris AI Microscopy Image Analysis x64 11.0.0 Software (Oxford Instruments Group, United Kingdom). Fluorescence intensities of desired *X*, *Y*, or *Z* regions of interest (ROIs) were calculated after background intensity subtraction.

### Red blood cell studies

The drawing of blood followed a protocol approved by the Institutional Review Board of Saint Louis University. All record keeping was in compliance regulations of the Health Insurance Portability and Accountability Act. Whole blood (40 mL) was collected *via* venipuncture from healthy human donors into heparinized syringes and centrifuged at 500*g* and 4 °C for 10 minutes. The plasma and buffy coat were removed by aspiration. The remaining RBCs were washed three times with a physiological salt solution (PSS) containing 21.0 mM tris(hydroxymethyl)aminomethane, 4.7 mM KCl, 2.0 mM CaCl_2_, 140.5 mM NaCl, 1.2 mM MgSO_4_, 5.5 mM glucose, and 0.5% (w/v) bovine serum albumin, at pH 7.4. RBCs were used for analysis within two days of drawing. When stored overnight, the RBCs were stored in PSS at 4 °C and washed three times in PSS the day of use. RBCs were diluted with PSS to a final hematocrit of 7% prior to injection into the device. All solutions were prepared the day of the experiment. For standard addition, 25 µM ATP standard stock solution was made in PSS buffer. Standard addition samples were prepared with 0 µL, 125 µL and 250 µL of the ATP standard stock solution respectively spiked in microcentrifuge tube each containing 1 mL of RBCs. PSS buffer was then used to dilute the RBCs samples to a total volume of 1.25 mL and make a final hematocrit of 7%. Measurements were performed in at least triplicate for each sample to generate the standard addition curve.

### ATP detection

For ATP detection, the channel downstream from the T-junction was placed over a PMT (R1527, Hamamatsu Photonics, Hamamatsu, Japan) housed in black box. For these studies, PSS buffer at a pH of 7.4 was used throughout as the carrier buffer for preparation and continuously pumped through the main channel of the device at 10 µL min^−1^. In addition, a four-port injector fitted with 1 µL rotor (Valco Instruments, Houston TX) was used for injection of a RBC plug through the main channel. The luciferin/luciferase reagent in the studies was prepared in a 10 : 1 ratio,^19^ with 34 mg mL^−1^ crude firefly lantern and 3.4 mg luciferin dissolved in 34 mL of PSS buffer at pH 7.4. The reagent is loaded into the 1 mL syringes and continuously pumped through the side channels of the device at varying flow rates (to vary the focusing widths). Flow injection analysis of the 1 µL plug of RBCs yielded peaks corresponding to the chemiluminescent signals. Peak heights were obtained from the RBC samples and the ATP-spiked RBC samples to quantify the unknown concentration of ATP present in the unspiked RBC samples using the method of standard additions. The peaks were smoothed in PeakFit (San Jose, CA using Savitsky–Golay filter at 0.5%) to filter out noise and ChromePerfect (Denville, NJ) was used for peak integration to get the peak height and area. The data presented here was prepared with OriginPro (Northampton, MA). The ATP concentrations are reported as averages (mean) and standard error of the mean (SEM).

## Results/discussion

The overall goal of this work was to create a 3D printed microfluidic device that could reproducibly focus RBCs to various degrees and quantitate the amount of ATP that the RBCs release in response to changes in the focusing widths. Long term, we would like to integrate the ability to manipulate RBCs in this manner with a 3D printed microfluidic-based transwell cell culture^20^ to investigate the role of the RBC and the blood–brain barrier in the onset of MS. We have previously reported a multi-modal 3D printed device that could measure ATP and NO release from RBCs under flow conditions.^19^ However, that PolyJet printed device used solid support to define the channel structure. Due to the need to clean out the solid support, the channel size was limited to 500 × 500 µm and the cells underwent very minor deformation, focused to a width of 200 µm due to the large channel size and relatively low flow rates (2.5 µL min^−1^). Effectively, we measured basal ATP and NO release under the flow conditions (average of 190 µM for ATP) and how those levels changed in response to reduced oxygen tension and blood storage conditions.^19^ We also used that device to investigate normoglycemic blood storage solutions^24^ and to evaluate a new recombinant–chemosynthetic biosensor for probing the surface of RBCs.^25^ In this work, we sought to produce devices with smaller channels that would enable deformation that was biologically relevant in a manner where we could easily change the focusing widths by simply changing the focusing channel flow rate and also measure the ATP release in close-to-real-time.

**Fig. 2:**
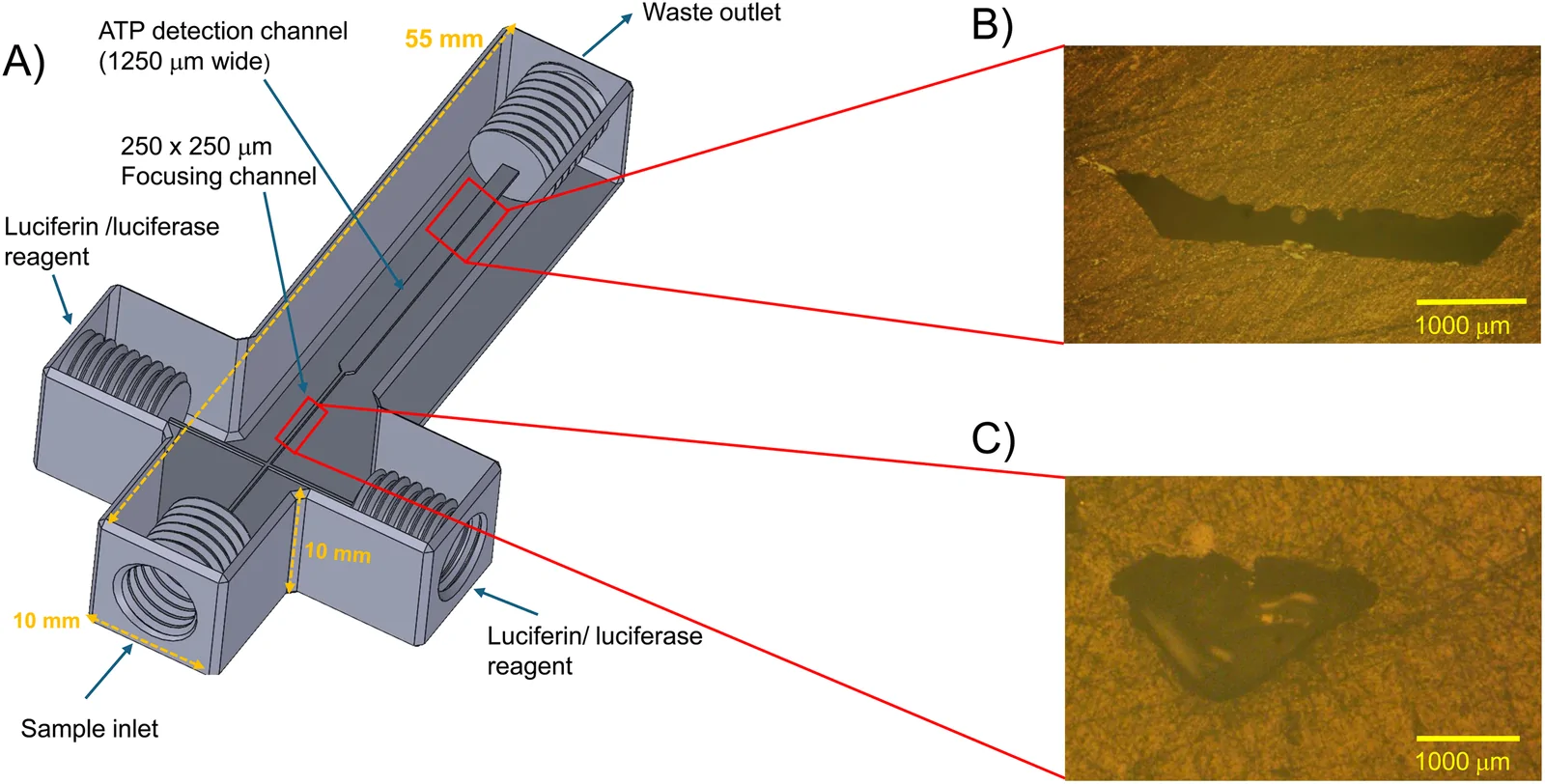
(A) CAD rendering of a microfluidic device used in this study with design dimensions denoted. The device contains threaded ports for fluidic fittings. (B) Cross-section of the expansion channel that serves as the ATP detection window area (measured average depth = 155 µm and width = 1200 µm, *n* = 6). (C) Cross-section channel around the focusing area (measured average depth = 170 µm and width = 240 µm, *n* = 9), just downstream of the mixing T.

PolyJet printing with liquid support and print-pause-print methodology^18^ was used to create the channels for the focusing device. Fig. 1A shows a CAD rendering of the bottom print layer. Once the channel layer is printed (and half of the threaded ports), the print is stopped, and liquid support is added to the channel (Fig. 1B). Printing is resumed for the top portion of the device (Fig. 1C) and after printing the liquid support is removed with air pressure. Fig. 2A shows the complete design and fluidic connections can be threaded into the device at the inlets and outlet. Focusing widths were obtained when a plug of RBCs and two streams of continuous flow of PSS buffer converge at the T junction to form a single stream of RBCs focused into a smaller width. Simultaneously, the device is robust enough to quantify ATP release when the ATP detection channel after the T junction is placed directly on the PMT window housed in a light-excluding black box. Device assembly and set-up take a few minutes. Ridges formed on the bottom of the device because of printing can be removed by polishing to increase the sensitivity of the device. The device is a simple tee, making the technology easily transferable from one research group to the order by means of sharing the SolidWorks file.

As can be seen in Fig. 2B, the cross-section of the device shows a non-square channel shape for the focusing and detection portion of the channel. The surface roughness at the top of the channel can be seen in Fig. S2. Both images show a trapezoidal shape, with the measured channel width and depth being smaller as compared to the initial design, with the bottom of the channel being narrower than the top. This is because the channel is printed open and the liquid support is only added once the channel portion of print is complete, with some of the print material bleeding into the channel structure as each layer is printed over time (making the channel narrower at the bottom). An average measured width of 240 µm and depth of 170 µm (*n* = 9) was obtained for the channels around the T junction (designed to be 250 × 250 µm, Fig. 2), while an average measured width of 1200 µm and depth of 154 µm (*n* = 6) was obtained for the mixing/expansion channel (designed to be 1250 × 250 µm, Fig. 2).

### Device characterization

**Fig. 3:**
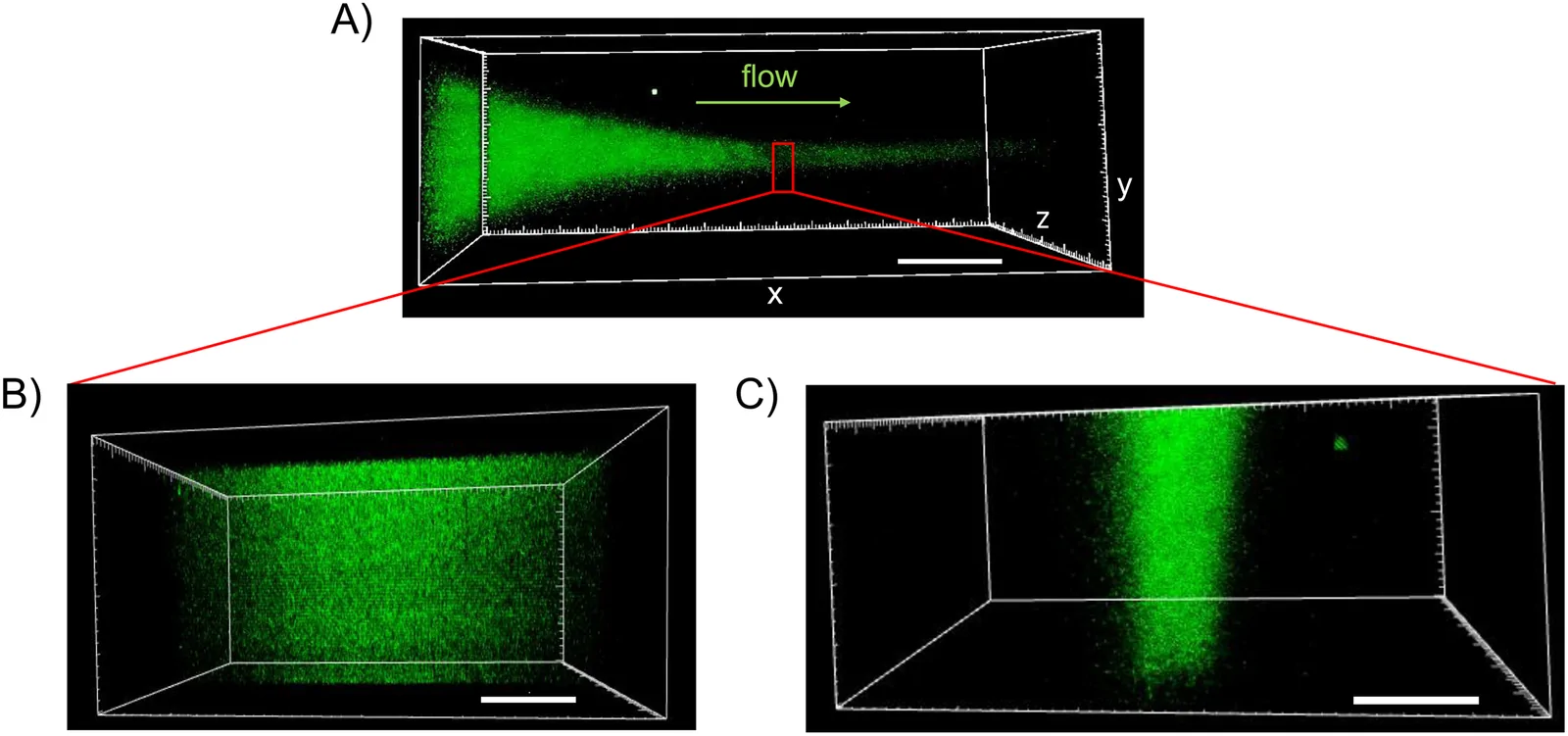
3D Confocal fluorescence imaging to examine the shape of focusing using a 10 µM fluorescein solution at 30 µL min^−1^. (A) Bottom-up view showing focusing in the *X* and *Y* dimension (a standard fluidic focusing image). (B) In the region denoted by the red box, fluorescent image showing flow throughout the *Z* plane, with the perspective being from the side of the channel. (C) In the region denoted by the red box, fluorescent image showing flow in the *Z* plane, with flow coming out of the device (towards the channel outlet). This image shows that the focused stream shape is similar to the flow channel shape. (scale bars: 100 µm for A, 50 µm for B and C).

Confocal fluorescence imaging was used to visualize the three-dimensional flow profile within the microfluidic channel from the bottom, side, and channel exit perspectives (Fig. 3). As illustrated in Fig. 3A, a 10 µM fluorescein solution was hydrodynamically pumped (10 µL min^−1^) to the T-junction, at which point a PBS solution was introduced through the side channels at 30 µL min^−1^. A reconstructed vertical cross-section of the focused stream obtained *via* confocal imaging, with the perspective being from the side of the channel (Fig. 3B), demonstrates a uniform flow profile extending stably from the T-junction to the outlet of the main channel. A z-stack image of the focusing stream as it exits the device (towards the channel outlet, Fig. 3C) confirms hydrodynamic focusing while also revealing the trapezoidal cross-sectional distribution of the focused stream that reflects the channel geometry.

**Fig. 4:**
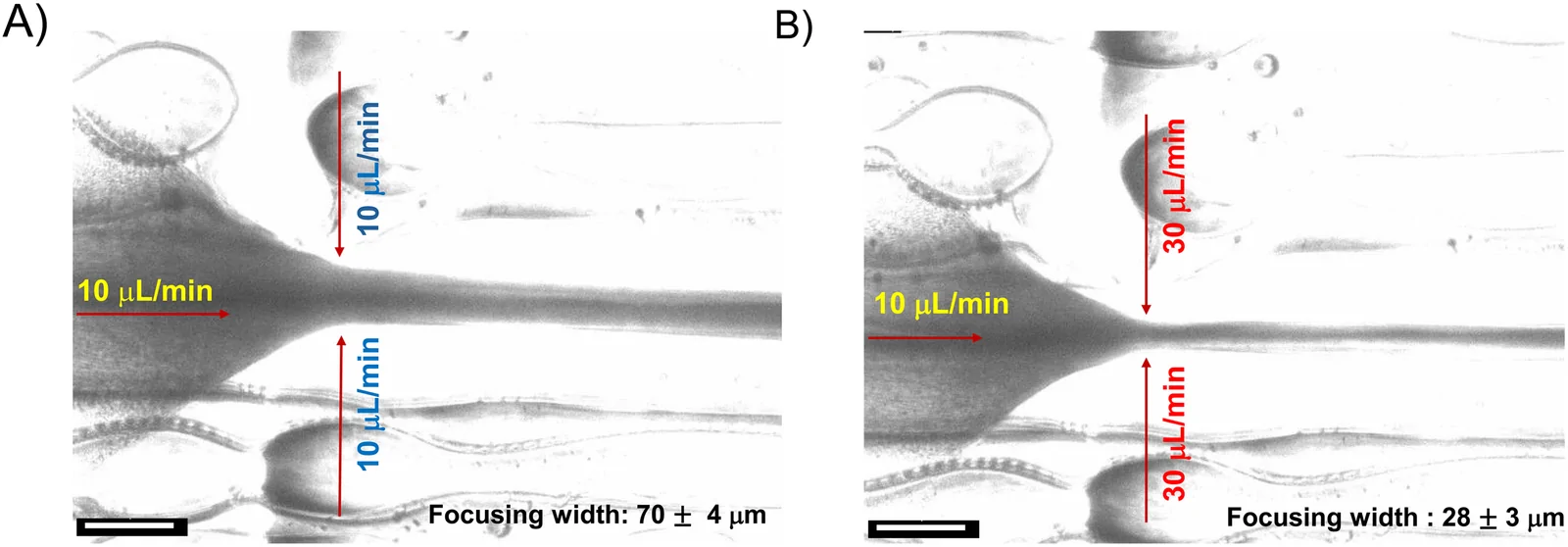
Hydrodynamic focusing of an RBC plug varying the sheathing flow rates (A) Bright field images of focusing RBCs at 10 µL min^−1^ with PSS buffer to achieve an average focusing width of 70 ± 4 µm (*n* = 16 different devices). (B) Bright field image of focusing RBCs at 30 µL min^−1^ resulted to an average focusing width at 28 ± 3 µm (*n* = 16 different devices). (scale bar: 100 µm).

RBCs are deformed as they transverse the microvasculature system.^26–28^ To study the focusing of RBCs in a 3D printed device, RBCs were pumped through the inlet of the optimized T design at a constant flow rate of 10 µL min^−1^, and PSS buffer was pumped through the sides channels at two distinct flow rates, either 10 µL min^−1^ (for minor deformation) or 30 µL min^−1^ (for more significant deformation). To study the changes in the focusing widths, inverted bright field microscopy was used to visualize the continuous flow of a RBC solution that was introduced in the main channel inlet at 10 µL min^−1^, with the sheathing flow rates changing from 10 µL min^−1^ to 30 µL min^−1^ (Fig. 4). Continuous flow of RBCs at 10 µL min^−1^ resulted in average measured RBC stream width of 70 ± 4 µm (*n* = 16). When the flow rate was increased to 30 µL min^−1^, the average measured RBC stream width decreased to 28 ± 3 µm (*n* = 16). As previously noted, the printed dimensions of channels around the T-junction were measured to be an average of 240 µm in width and 170 µm in depth (*n* = 9). When focusing the RBC stream, this leads to a focusing cross-section of 70 (width) × 170 (depth) µm when 10 µL min^−1^ is applied to the side channels (Fig. 4A) or 28 × 170 µm when 30 µL min^−1^ is applied to the side channels (Fig. 4B).

Smaller cross-sectional areas provide a good mimic to exert pressure on RBCs as they traverse through them with the application of flow.^12^ A paper previously published from our group investigated a T-device to measure basal ATP and NO release from RBCs using a large channel (500 × 500 µm) and low flow rates of luciferin/luciferase (2.5 µL min^−1^), with very little focusing occurring. In that paper, we explored different designs and found that a double T design offers better calibration sensitivity compared to a single T design.^19^ In this study, an increase in the sensitivity of the device is necessary to detect ATP from RBCs because of the relatively higher linear velocities (from higher flow rates and smaller channels). This was done by increasing the channel width after the focusing area of the device, 10 mm downstream from the T-junction. This helped to decrease the linear velocity in that area (which sits over the PMT), leading to more time for mixing and reaction as well as more time for the PMT to detect the emitted photons. As mentioned earlier and shown in Fig. 2, the average measured width of the printed channels increased from 240 µm with the channels around the T-intersection to 1200 µm in the expansion region.

The LOD and calibration sensitivity for an ATP calibration curve were employed to determine the effect of including this wider channel for mixing and increased residence time. This was done with ATP concentrations ranging from 0.05 to 2.5 µM. The T-device shown in Fig. 2 was compared to one that is identical except for the channels being the same width throughout (no expansion region). Using the device without the expansion region and a 10 µL min^−1^ focusing flow rate led to a LOD of 73 nM and a sensitivity of 0.5886 V µM^−1^, while use of a 30 µL min^−1^ flow rate led to a LOD of 286 nM and a sensitivity of 0.2046 V µM^−1^. To enhance the sensitivity, we increased the channel width to 1200 µm downstream of the T to decrease the linear velocity and give more time to the deformed RBCs/released ATP to mix with the luciferin/luciferase reagent over the PMT and increase signal integration time. This greatly improved the sensitivity and LOD. The LOD of the device with the expansion was found to be 17 nM at 10 µL min^−1^ and 46 nM at 30 µL min^−1^. A similar trend was seen with sensitivity, with the 10 µL min^−1^ leading to a sensitivity of 2.256 V µM^−1^, (approximately 4.5-fold improvement) and the 30 µL min^−1^ resulting in a sensitivity of 0.3645 V µM^−1^ (approximately 1.8-fold improvement). A comparison of these determined values is shown in Table S1.

### Detection of ATP release

**Fig. 5:**
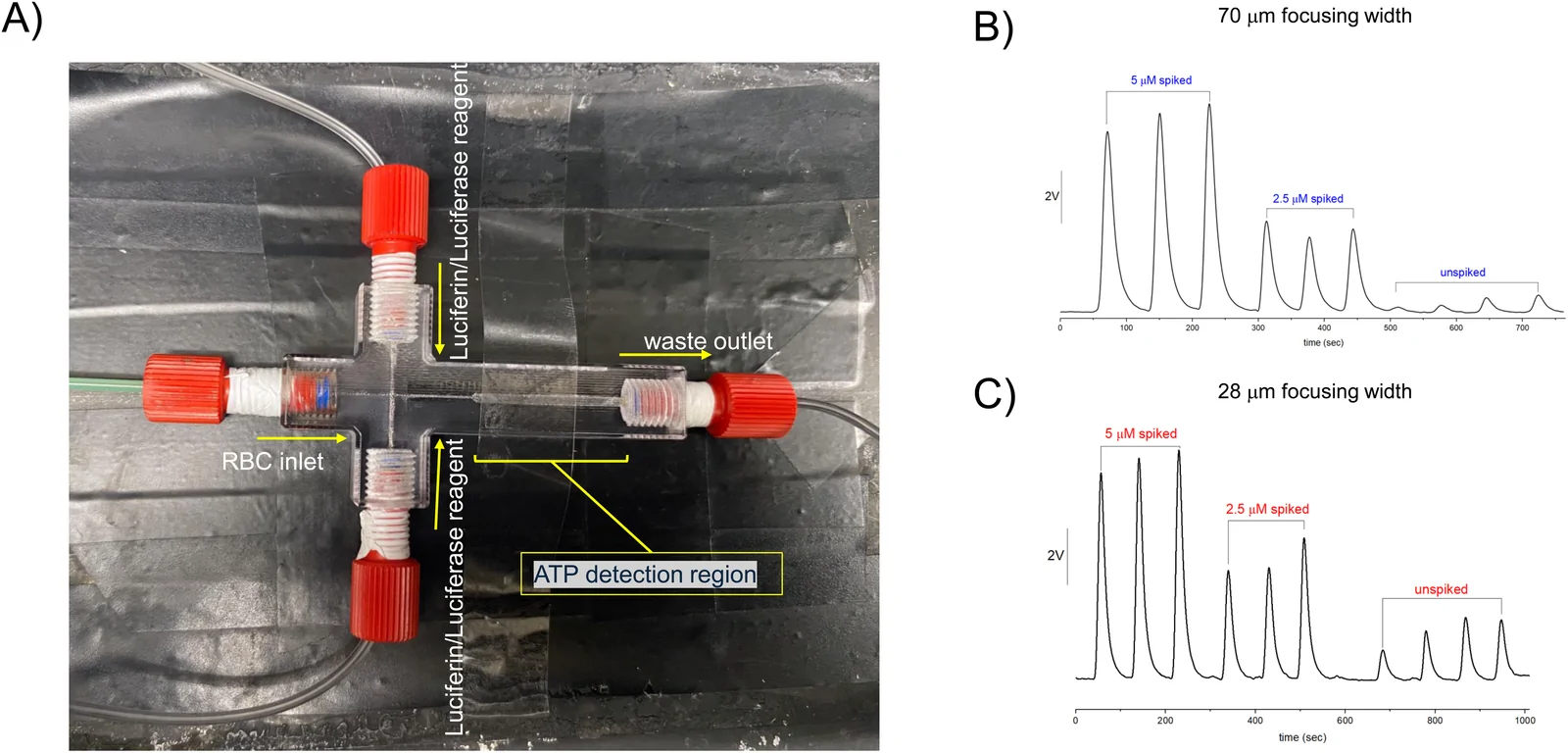
Chemiluminescent detection of ATP release from RBCs, comparing the 2 different focusing widths. (A) 3D printed device with mixing channel taped over PMT that is housed in a black box. Peaks were obtained using standard addition method where a plug of 7% RBCs was injected *via* a 4-port to the main channel at a constant flow rate of 10 µL min^−1^ and the side channel flow rate varied from (B) 10 µL min^−1^ (70 µm average focusing width), and (C) 30 µL min^−1^ (28 µm average focusing width).

The optimized device was used to quantitate the changes in ATP release from RBCs that result from changing the focusing widths. This was done by injecting a 1 µL plug of RBCs through the main channel inlet at 10 µL min^−1^ while luciferin/luciferase reagent was pumped through each of the side channels at the two flow rates (either 10 or 30 µL min^−1^). To maximize the capture of the photons emitted from the chemiluminescence reaction that occurs after the side stream of luciferin/luciferase focuses the central stream of RBCs (reacting with any released ATP), the microchannel region after the intersection is placed on the opening of the PMT window such that the expansion region also sits over the PMT (Fig. 5). To account for any matrix effects, the method of standard additions was utilized. For each standard addition/quantitation experiment, 3 sample vials with 1 mL of RBCs were prepared, 2 of which were spiked with ATP. The syringe pump for side channels was first set to 10 µL min^−1^ while keeping the main channel flow rate constant at 10 µL min^−1^ (70 µm focusing width). Triplicate measurements were taken for each of the samples and average peak heights were determined. Between each injection, the 4-port was thoroughly rinsed with PSS buffer to ensure no RBCs remained. Fig. 5B shows the peaks when a plug of 7% RBCs for the spiked and unspiked samples were injected into the microfluidic device at 10 µL min^−1^. It can be observed that the peaks for unspiked samples relative to spiked samples are smaller, which is due to low deformation because of reduced ATP release. On the other hand, the peaks observed at 30 µL min^−1^ (28 µm focusing width, Fig. 5C) were relatively larger due to increased ATP release.

**Fig. 6:**
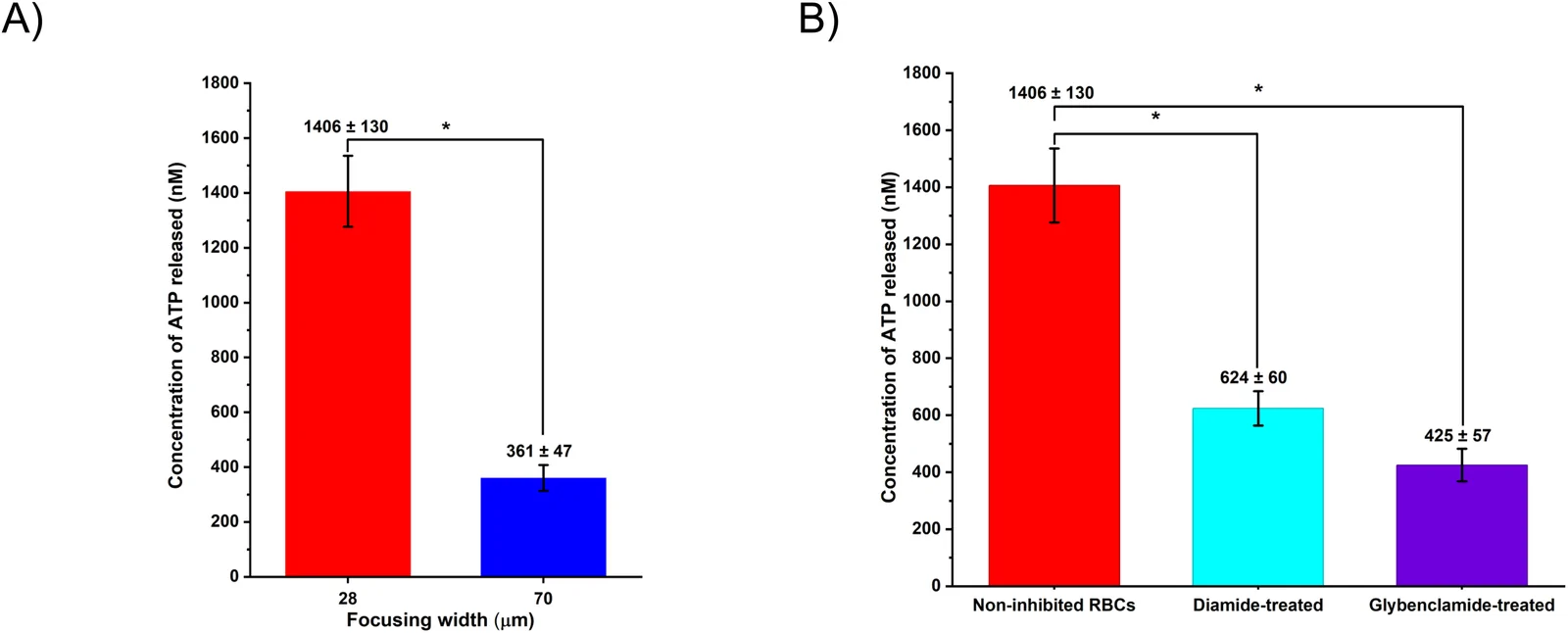
ATP release as a function of focusing width (ATP concentrations in both figures reported as mean ± SEM). (A) Bar graph showing that ATP release from healthy RBCs increases significantly (at 95% confidence level) when the focusing width changes from 70 to 28 µm (*n* = 23 donations). (B) Using a 28 µm focusing width, inhibition studies were carried out with diamide-treated RBCs (*n* = 7) and glybenclamide-treated RBCs (*n* = 7). In each case, the inhibitor samples gave a significantly reduced amount of ATP release (at 95% confidence level).

Fig. 6 shows the compilation of ATP release experiments for various healthy RBC samples. ATP release from healthy RBCs has been reported to be in the nanomolar range.^29^ Several healthy RBC samples were analyzed with the optimized device. As can be seen in Fig. 6A, operating a 10 µL min^−1^ for the focusing steam (70 µm focusing width) led to an average ATP release of 361 nM (*n* = 23) of ATP. For studies where the focusing rate was at 30 µL min^−1^ (28 µm focusing width), an average of 1.4 µM (*n* = 23) of ATP release was determined. This clearly shows that decreasing the focusing width deforms RBCs in a reproducible manner, leading to a measurable and significantly different amount of ATP release. In studies where the release of ATP from RBCs is determined, it is common to use different pharmacological agents to selectively reduce the release of ATP. This is done to ensure that differential release and deformation are possible and well as to ensure a lack of cell lysis (if lysis were present, no changes in the response would be seen). For the inhibition studies, diamide, a RBC-stiffening agent and glibenclamide, a CFTR inhibitor, were employed. Diamide, affects the cytoskeletal membrane by creating disulfide bonds on spectrin proteins, making the RBC more rigid.^30^ Using the higher focusing flow rate of 30 µL min^−1^ (28 µm focusing) and incubating the RBCs with 100 µM diamide for 15 minutes before analysis led to an average of 624 nM ATP being released from the RBCs (*n* = 7), a 2.3 reduction in the average ATP release (see Fig. 6B). Glibenclamide inhibits the CFTR, which regulates the ion channel that allows ATP release as a result of deformation.^31^ Glibenclamide-treated RBCs showed a similar trend, with RBCs incubated with 100 µM glibenclamide for 15 minutes resulting in an average ATP release of 425 nM (*n* = 7), a 3.3 reduction compared to the average ATP release from untreated RBCs. Clearly, this device can measure changes in ATP release from RBCs as a function of the focusing width without issues from cell lysis.

## Conclusion

In this study, we designed a 3D-printed microfluidic device to simultaneously focus RBCs and measure the subsequent ATP release, with deformation changes being modulated by simply adjusting the focusing flow rates. We overcame the challenge of printing smaller channels with PolyJet and solid support by employing the print-pause-print technique using a liquid support material. The device was characterized and optimized to enhance the sensitivity and limit of detection, as well as to determine the concentrations of ATP released from healthy RBCs at varying focusing widths including inhibition studies. Real-time analysis of deformation-induced ATP release from RBCs mimics *in vivo* interactions that RBCs experience as they transverse the microvasculature *in vivo*. In addition, these 3D printed devices could possibly be used as a diagnostic tool for those with RBC deformability issues. Future work will entail using this approach to quantitate the ATP release from diseased RBCs and to integrate this approach with microfluidic transwell devices^20^ to result in a true blood–brain barrier mimic where deformation of RBCs can be easily varied. We are also investigating 3D printing approaches to focus the cells in all 3 dimensions.

## Conflicts of interest

There are no conflicts to declare.

## Supplementary Material

AY-018-D6AY01598K-s001

## Data Availability

The data for this paper is included in the manuscript and supplementary information (SI). Any other data requests are available from the corresponding author upon reasonable request. Supplementary information: CAD dimensions, further characterization and standard addition plots. See DOI: https://doi.org/10.1039/d6ay01598k.
