# Supplementary material for: PolyJet 3D-printed microfluidic device that integrates hydrodynamic focusing of red blood cells and detection of ATP release
Source: Anal Methods. 2026 Sep 18;18(40):9325–33. doi: 10.1039/d6ay01598k (PMC13588292; doi:10.1039/d6ay01598k)
Supplement: Supplementary file 1 [file AY-018-D6AY01598K-s001.pdf]

Supplementary Information (SI) for Analytical Methods.
This journal is © The Royal Society of Chemistry 2026

Supplementary Information for:

PolyJet 3D-Printed Microfluidic Device that Integrates Hydrodynamic Focusing of Red Blood Cells and Detection of ATP Release

Mariama Singhateh,[a] Samuel Azibere,[a] Randy S. Spague,[b] Ajith Karunarathne,[a] Sashima Liyanage,[a] and R. Scott Martin[a,c*]

[a] Department of Chemistry, Saint Louis University
[b] Department of Pharmacological and Physiological Science, Saint Louis University, 1402 S. Grand Boulevard, Saint Louis, MO 63103
[c] Center for Additive Manufacturing, Saint Louis University

*corresponding author:
Dr. R. Scott Martin
3501 Laclede Ave
St. Louis, MO, USA 63103
+1 314-977-2836
scott.martin@slu.edu

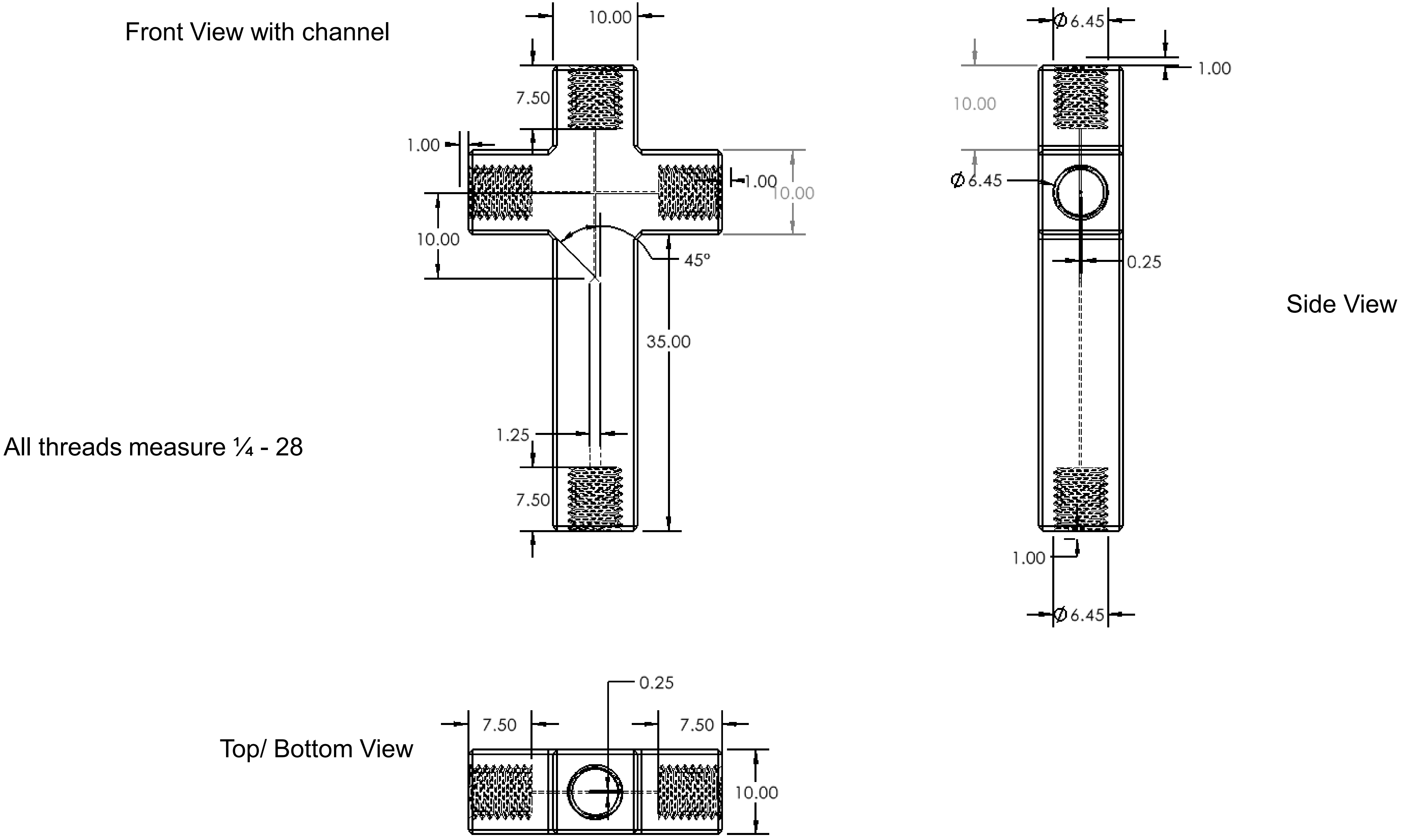

Fig. S1 CAD Design for optimized T device. All dimensions are in millimeters (mm).

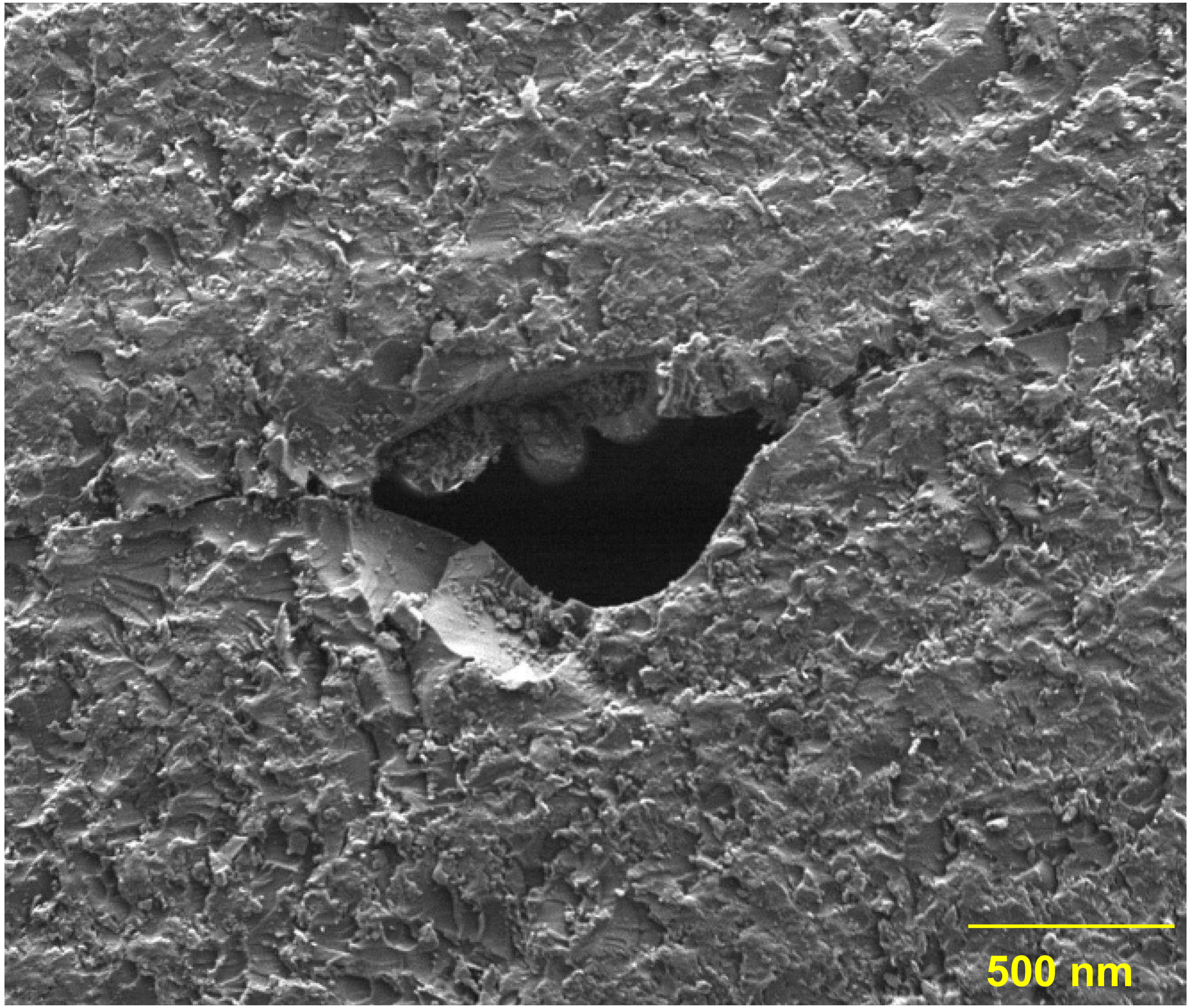

Fig. S2 SEM of the dissected device close to the T intersection. This image shows the features at the top of the channel (due to printing over the liquid support), as well as the channel shape.

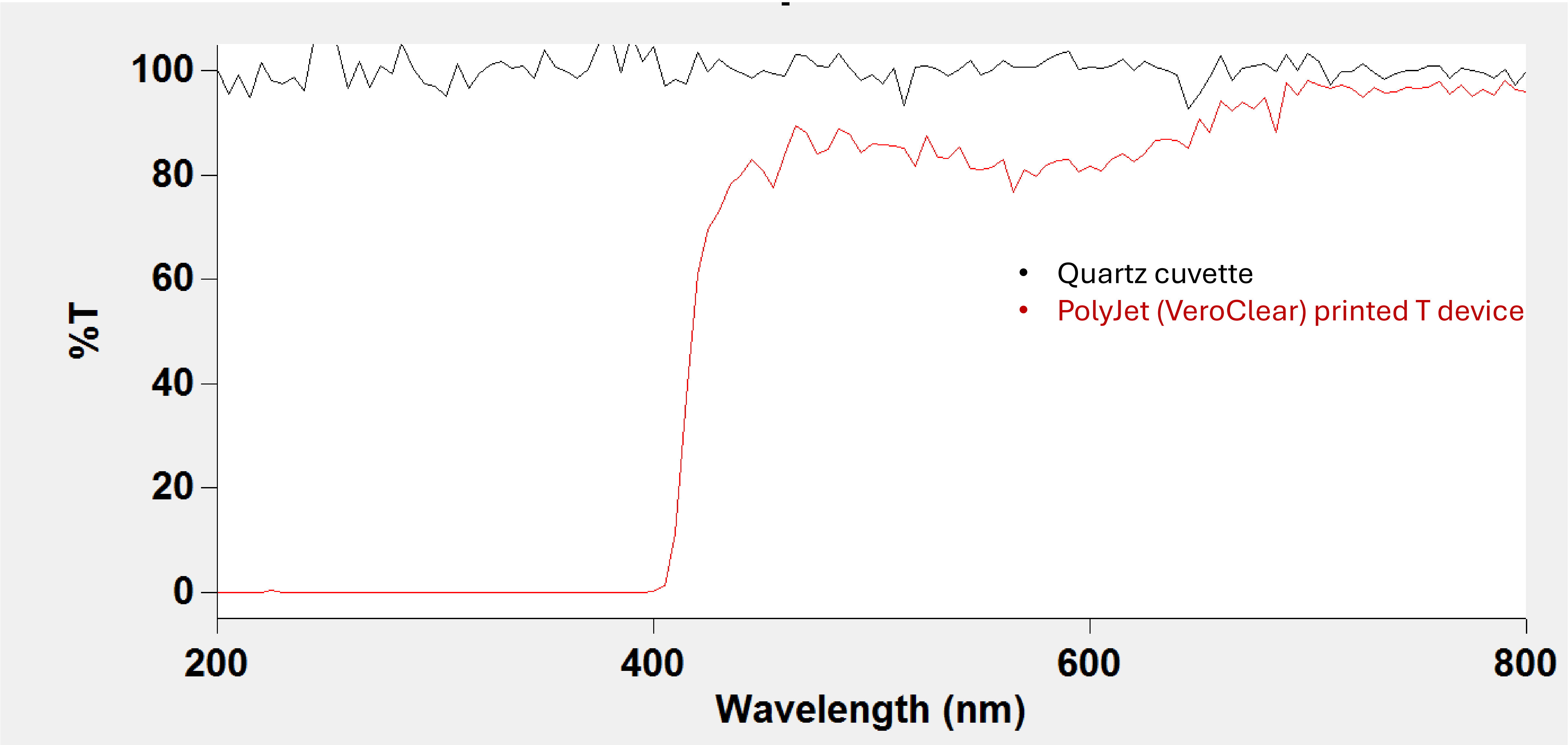

Fig. S3 UV-Vis transmittance spectrum of the 3D printed microfluidic T device compared to a 10 mm quartz cuvette.

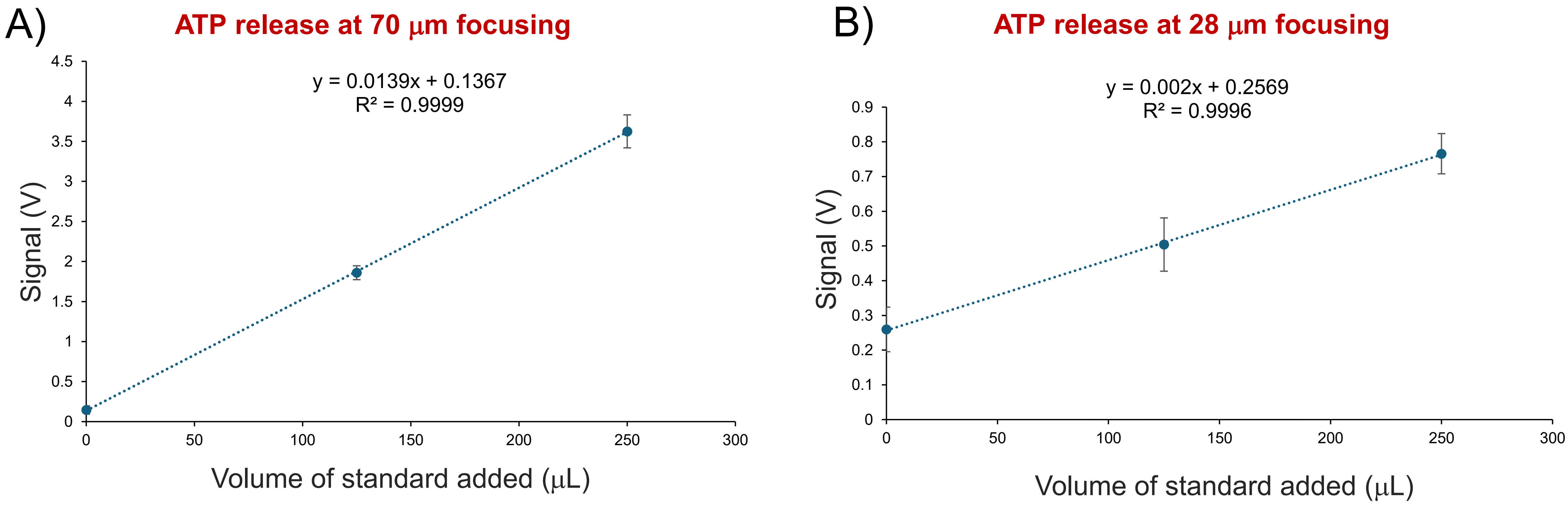

Fig. S4 Representative standard addition plots at each of the flow rates used in this study. A) 10 µL/min, 70 µm focusing and B) 30 µL/min, 28 µm focusing.

	10 μL/ min without expansion	10 μL/min with expansion		30 μL/min without expansion	30 μL/min with expansion
Sensitivity (V μM^{-1})	0.5886	2.256		0.2046	0.3645
LOD (nM)	73	17		286	46

Table S1. Comparison of the calibration sensitivity and LOD for the focusing device with and without the expansion mixing channel.
